# Tunable Infrared Emissivity in Multilayer Graphene by Ionic Liquid Intercalation

**DOI:** 10.3390/nano9081096

**Published:** 2019-07-31

**Authors:** Liyuan Zhao, Renyan Zhang, Chuyun Deng, Yuanxi Peng, Tian Jiang

**Affiliations:** 1 State Key Laboratory of High Performance Computing, National University of Defense Technology, Changsha 410073, China; 2 College of Advanced Interdisciplinary Studies, National University of Defense Technology, Changsha 410073, China; 3 College of Arts and Science, National University of Defense Technology, Changsha 410073, China

**Keywords:** graphene, ionic liquid, intercalation, flexible, infrared emissivity

## Abstract

Controllably tuned infrared emissivity has attracted great interest for potential application in adaptive thermal camouflage. In this work, we report a flexible multilayer graphene based infrared device on a porous polyethylene membrane, where the infrared emissivity could be tuned by ionic liquid intercalation. The Fermi level of surface multilayer graphene shifts to a high energy level through ionic liquid intercalation, which blocks electronic transition below the Fermi level. Thus, the optical absorptivity/emissivity of graphene could be controlled by intercalation. Experimentally, the infrared emissivity of surface graphene was found to be tuned from 0.57 to 0.41 after ionic liquid intercalation. Meanwhile, the relative reflectivity R_v_/R_0_ of surface graphene increased from 1.0 to 1.15. The strong fluorescence background of Raman spectra, the upshift of the G peak (~23 cm^−1^), and the decrease of sheet resistance confirmed the successful intercalation of ionic liquid into the graphene layers. This intercalation control of the infrared emissivity of graphene in this work displays a new way of building an effective thermal camouflage system.

## 1. Introduction

All matter at a temperature above absolute zero emit infrared waves, the intensity of which is dependent on the temperature (T) and infrared emissivity (ε) of the surface materials [1]. This is widely used nowadays for night vision [2], infrared source [3,4], and temperature measurement [5]. Since thermal cycling is usually quite slow [6], mere temperature control could not satisfy the need for the further development of the infrared source [3,4], thermal management [7], and thermal camouflage [6,7,8]. Thus, it would be of great significance to controllably tune infrared emissivity (ε) no matter whether for fundamental research or for real applications. Recently, it was found that infrared emissivity could be modulated in situ through the design of surface structure [7], controlling of carrier density [7,8] or phase transition by external stimuli [6]. These results indicate that proper tuning of the electronic structure of the surface materials is an effective way to modulate infrared emission. However, these materials are usually on a rigid substrate and the tuning range is quite limited [7]. A flexible material with a tunable infrared emissivity is highly desirable.

Due to linear band dispersion, graphene has a very broad band optical absorption/emissivity (Kirchhoff’s radiation law shows that the infrared absorption and emissivity are equal to each other at thermodynamic equilibrium) [3,9,10,11,12]. However, the interband electronic transition below the Fermi level is blocked in graphene due to the Pauli exclusion principle. Thus, doping, which shifts the Fermi level to a high energy level [7,9,10,13,14], yields tunable optical absorbance [9,10] or light emission [7,11,15,16] of graphene in a very broad spectral range from visible to far infrared frequencies. Unfortunately, the absorption/emissivity of monolayer graphene is limited to 2.3% by the fundamental fine structure constant [12], which is obviously not acceptable for real applications. Increasing the number of graphene layers increases the optical absorption/emissivity [17,18,19], which is an achievable way to increase the modulation depth of absorption/emissivity. However, electrostatic doping, the common way of doping in monolayer graphene, is not possible for tuning the interband transition for multilayer graphene, because of the shielding effect of the surface layers. Recently, intercalation has been demonstrated to be an effective way to dope multilayer two-dimensional (2D) materials [17,18,19,20,21,22,23,24,25,26,27]. The intercalation process is reversible and compatible with the current semiconductor fabrication process [17,19,22,26,27], which makes it very promising to tune the infrared emissivity of multilayer graphene.

**Figure 1:**
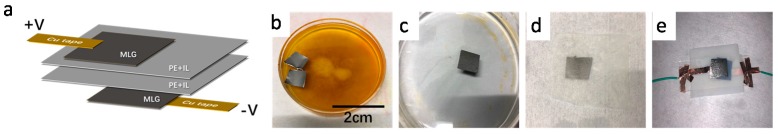
Device fabrication. (**a**) Schematic of the infrared multilayer graphene devices. (**b**) The optical image of the etching process. (**c**) The optical image of the deionized water washing process. (**d**) The optical image of multilayer graphene on a porous polyethylene membrane. (**e**) The optical image of the final device.

In this work, by intercalating nonvolatile ionic liquid [DEME][TFSI] (98.5%, diethyl methyl (2-methoxyethyl)—ammonium bis (trifluoromethyl sulfonyl) imide, Sigma-Aldrich, Catalog No.727679, St. Louis, MO, USA) into multilayer graphene layers, we demonstrated a flexible thermal surface, which can electrically control the infrared emission without changing the surface temperature. Upon ionic liquid intercalation, the Fermi energy of graphene upshifts from the Dirac point to a high energy level, causing the emissivity of graphene to reduce from 0.57 to 0.41. The increase of the infrared relative reflectivity (R_v_/R_0_ of graphene with the sample on copperplate is increased from 1.0 to 1.15) confirms the decrease of emissivity. The in-situ Raman spectra with an electronic voltage above 3 V have a strong fluorescence background and an upshift of the G peak of about 23 cm^−1^, which indicates a strong doping effect of graphene layers with intercalation. In addition, the sheet resistance of graphene is reduced from 11 to 4 Ω/□ after intercalation due to the charge transfer between the ionic liquid and graphene layers. What is more, the intercalation process is fast and reversible; the film is light, flexible, and thin (<20 μm); and the four-layer architecture device architecture (see Figure 1a) is compatible with modern roll-to-roll transfer processes. This makes the device in our work a promising material for designing tunable thermal camouflage systems.

## 2. Materials and Methods

**Sample preparation**: The multilayer graphene film with a thickness of about 50–70 nm used in this work is on nickel (Ni) foil (Changzhou Carbon Time Technology Co., Ltd., Jiangsu, China). The supporting Ni foil was etched away in saturated iron chloride (FeCl_3_) aqueous solution (Aladdin Industrial Corporation, Shanghai, China), resulting in a free-standing multilayer graphene film on the solution surface (as shown in Figure 1b). Then, we transferred the multilayer graphene film to deionized water to remove the residual FeCl_3_, as shown in Figure 1c. After that, the multilayer graphene film was transferred onto the porous polyethylene membrane (Fisher Science, Catalog No.14-100-139, Hampton, NH, USA), which was infrared transparent and flexible. In order to remove the remaining water, the multilayer graphene on the porous polyethylene membrane (Figure 1d) was baked in an oven at 50 °C. Conductive silver paste was painted on the edge of the multilayer graphene film as contact electrodes. To fabricate the infrared tunable devices, we attached two graphene coated porous polyethylene membranes together, with the multilayer graphene facing outside (Figure 1e). Then, we injected about 50 μL nonvolatile ionic liquid [DEME][TFSI] (98.5%, diethyl methyl (2-methoxyethyl)—ammonium bis (trifluoromethyl sulfonyl) imide, Sigma-Aldrich, Catalog No.727679, St. Louis, MO, USA) between the two porous polyethylene membranes, which can be used to hold the ionic liquid. Figure 1e shows the optical image of the final device. In this structure, multilayer graphene operates as both electrically conductive electrodes and optical medium.

**Characterization of ionic liquid intercalated multilayer graphene**: The four-layer architecture structure of the multilayer graphene device makes it possible to carry out the in- situ characterization under different voltage bias. In this work, a Keithley 2450 source meter (Tektronix, Beaverton, OR, USA) was used to apply voltage bias between two multilayer graphene layers to control the intercalation process. In-situ Raman measurements were conducted with a XperRam Compact Raman spectrometer (Nanobase, Seoul, Korea), with excitation laser wavelength of 532 nm and laser power of 0.5 mW. The sheet resistance of the multilayer graphene under different intercalation voltage bias was measured through another Keithley 2636B source meter (Tektronix, Beaverton, OR, USA) using the four-point method. In order to analyze the change in the infrared emissivity, the reflection of the multilayer graphene device was measured with the device on a polished copper plate (thickness about 1 mm), which is described in detail below. The in-situ reflection measurement under voltage bias was carried out on a U-4100 spectrophotometer (Hitachi, Tokyo, Japan) with a wavelength range from 400 to 2000 nm.

**Figure 2:**
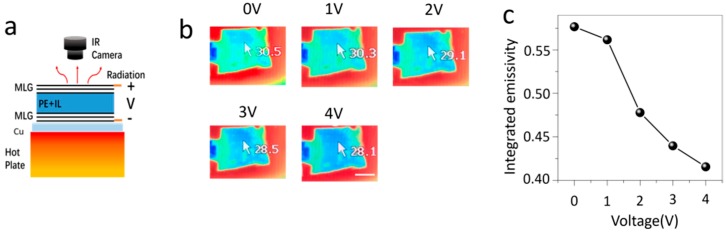
Thermal imaging of the ionic liquid intercalated multilayer graphene device. (**a**) Schematic of the thermal imaging process. In this process, the device is put on a polished copper plate and then on a hot plate. (**b**) The thermal images of the multilayer graphene device under different intercalation voltage bias. The scale bar in (**b**) is 1 cm. (**c**) The change of the integrated emissivity with intercalation voltage bias.

**Thermal imaging**: During the thermal image measurement, the multilayer graphene device was put on a polished copper plate (the emissivity was about 0.1), then the copper plate with the device was put on a hot plate (as shown in Figure 2a). A thermocouple was used to measure the temperature of the surface multilayer graphene. The temperature of the hot plate was adjusted to keep the surface multilayer graphene temperature at 35 °C, with the surrounding temperature at 20 °C. The thermographs of the multilayer device at different intercalation bias voltages were recorded using a Tix500 thermal camera (Fluke, Everett, WA, USA), with a constant emissivity of 1.

## 3. Results and Discussion

For thermal imaging measurements, the multilayer graphene device was put on a polished copper plate (Figure 2a), which prevented the transmission of background infrared emission [8,17,22]. Due to the low infrared emissivity of polished copper substrate (~0.1) and the porous polyethylene membrane (<0.1), the thermal radiation was mainly from the surface multilayer graphene. To analyze the infrared behavior of the multilayer graphene surface, the device with the polished copper substrate was put on a hot plate. A thermocouple was used to monitor the temperature of the surface graphene. The temperature of the hot plate was adjusted to keep the surface graphene temperature at 35 °C. A Tix500 thermal camera (Fluke, Everett, WA, USA) was adopted to record the thermal images (emissivity was set as 1) at different intercalation bias voltages between 0 and 4 V. The voltages bias range is mainly limited by the electrochemical window of the ionic liquid at room temperature. Additionally, for bias voltages larger than 5 V, the surface graphene l turns to dark black and cannot be reversed. This is likely due to the oxidation of surface graphene, which usually becomes sensitive after heavily doping. Figure 2b shows that the infrared temperature of the device is reduced from 30.5 °C at 0 V to 28.1 °C at 4 V, although the surface temperature has not changed at 35 °C. This implies the emissivity of the device is suppressed by ionic liquid intercalation.

At thermodynamic equilibrium, the infrared emission energy is described by the Stefan−Boltzmann law, P = εσT^4^, where ε is the emissivity of the surface, σ is the Stefan−Boltzmann constant, and T is the temperature of the surface. Thus, the integrated emissivity ε of the surface could be determined by ε = ε_I_(T_I_/T)^4^, where ε_I_ is the emissivity used for thermal imaging, T_I_ is the infrared temperature, and T is the real temperature measured by thermocouple. Thus, the integrated emissivity for different intercalation bias voltages could be determined from the thermal images. The integrated emissivity at different bias voltages is summarized in Figure 2c, and can be tuned from 0.57 to 0.41 through intercalation. What is necessary to mention is that the modulation of infrared emissivity is reversible and the switch time between different states is very fast with a response time less than 1s.

**Figure 3:**
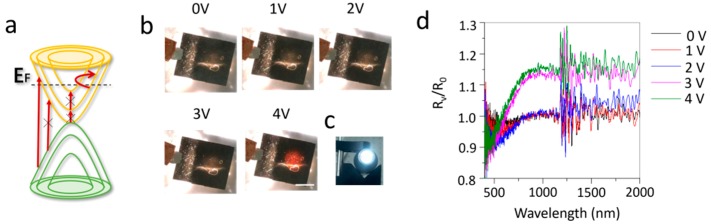
The in-situ reflection of ionic liquid intercalated multilayer graphene device. (**a**) Electronic band structure of doped multilayer graphene and possible electronic transitions. × means that this transition is blocked due to the upshift of Fermi level. (**b**) Photographs of the devices under different voltage bias, with a xenon lamp (**c**) under the device. The scale bar in (**b**) is 1 cm. (**d**) Reflectance spectra (R_V_/R_0_) of the multilayer graphene device on polished copper plate under different voltage bias.

Due to the ionic liquid intercalation, the Fermi level of graphene shifts to a higher energy level. The electronic transition below the Fermi level is suppressed due to Pauli blocking (Figure 3a), resulting in a decrease of the emissivity/absorption. Since the polished copper plate has a very high infrared reflectivity (~100%) and the porous polyethylene membrane is infrared transparent, the transmission of the multilayer graphene device on polished copper plate is 0. Thus, the emissivity of the surface multilayer graphene can be written as ε = α = 1 − R, where ε, α and R are the emissivity, absorption and reflectivity of surface multilayer graphene on polished copper plate. Figure 3d presents the in-situ reflectance (R_V_/R_0_) measurement of the multilayer graphene device on polished copper plate. The reflectance measurement shows that above 3 V there is an obvious increase of reflectance. This implies that the decrease of absorption/emissivity above 3 V, is consistent with Figure 2c. In addition, we found that the reflectance below 500 nm remained almost unchanged with intercalation. This indicates that ionic liquid intercalation is more efficient for modulating the optical response at long wavelength range. Additionally, the multilayer graphene device was put on a xenon lamp (Figure 3c), which produced a round shape of white light illumination. With the voltage bias between two multilayer graphene layers increasing from 0 to 4 V, the light illumination became visible. In other words, the transmittance (absorption) of multilayer graphene is increased (decrease), due to the increase of Fermi energy through ionic liquid intercalation. However, the light illumination on the graphene device is red not white, which indicates that the optical modulation is more effective for long wavelengths, such as the infrared range, agreeing with the reflectance measurement (Figure 3d).

**Figure 4:**
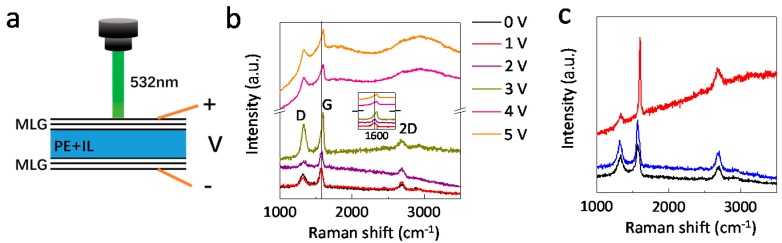
In-situ Raman measurements of the ionic liquid intercalated multilayer graphene device. (**a**) Schematic of the in-situ Raman measuring process. (**b**) Raman spectra of the surface multilayer graphene under different bias voltages. The inset shows the zoom in for G peak. (**c**) Raman spectra of pristine (black), intercalated (red) and deintercalated (blue) surface multilayer graphene.

The modulation of infrared emissivity is clearly due to the intercalation of the ionic liquid [DEME][TFSI] into the graphene layers. To further characterize the intercalation process of surface multilayer graphene, in-situ Raman measurement was carried out (Figure 4a). Figure 4b presents the Raman spectra for surface graphene under different bias voltages. For a pristine multilayer graphene, there are three Raman modes: D (1321 cm^−1^), G (1580 cm^−1^), and 2D (2688 cm^−1^) modes, consistent with previous research [28,29,30]. The D Raman modes indicates the defect in graphene, which is probably induced by the etching and transfer process [22]. For an intercalation bias voltage below 2 V, the Raman spectrum remains similar to that of the pristine sample. However, when the applied voltage is above 3 V, the intensity of the G mode and D mode is increased significantly and the G mode is found to shift from 1580 to 1603 cm^−1^ with the bias voltage increase to 3 V. The increase of the G mode intensity is an indication of the doping effect through intercalation [17,22]. While, the upshift of 23 cm^−1^ of the G mode implies the successful intercalation [27]. The increase of D mode intensity shows the increase of defects in the graphene layers during the intercalation process, which enhances the intercalation process. For a bias voltage above 4 V, a strong fluorescence background appears with the diminishing of the 2D Raman modes. This further confirms the strong doping effect of intercalation. After removal of the applied voltage, the surface graphene shows a similar Raman spectrum to the pristine sample (Figure 4c). In other words, the intercalation process is reversible.

**Figure 5:**
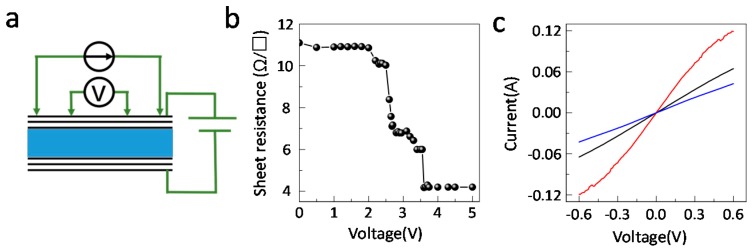
The sheet resistance of ionic liquid intercalated multilayer graphene. (**a**) Schematic of the in-situ sheet resistance measurements system. (**b**) The dependence of the sheet resistance on the intercalated bias voltage. (**c**) I-V curves of pristine (black), intercalated (red) and deintercalated (blue) multilayer graphene.

The sheet resistance under the intercalated bias voltage of multilayer graphene was also measured by the four-point resistivity method (Figure 5a). The weak Van der Waals forces between the graphene layers allows atom or small molecules to intercalate into the Van der Waals gap [20]. In this case, the anions/cations of the ionic liquid intercalate into the layers under a voltage bias. As a result, the charge density on the graphene increases significantly and the sheet resistance of the multilayer graphene has a sharp drop from 11 Ω/□ below 2 V to 4 Ω/□ above 3.5 V (Figure 5b). This is consistent with the Raman measurements. These results suggest that there is a threshold bias voltage around 2 V. At a voltage below 2 V, the ions accumulate at the graphene—ionic liquid interface while above 2 V, the ions are intercalated onto the surface graphene layers. The variation of the integrated emissivity and sheet resistance is similar. However, the integrated infrared emissivity could also be modulated below 2 V (Figure 2c). This is attributed to the electrostatic doping effect of the accumulating ions at the surface graphene sample, resulting in a small infrared emissivity modulation. Additionally, the intercalation process is reversible, with the deintercalated graphene sample having similar I-V curves to that of pristine graphene samples (Figure 5c).

In addition, we noted that the change of the infrared temperature of the surface multilayer graphene started at the edge and then propagated across the device at the very beginning. However, the intercalation process induces defects on sthe urface multilayer graphene, which was demonstrated by the increase of D peak intensity in the in-situ Raman spectra (Figure 4b). Thus, for the following cycle, the change of the infrared temperature of the surface multilayer graphene alters homogeneously across the whole sample, i.e., defects are induced across the whole sample. To test the long-term stability of the multilayer graphene device, we tested the infrared and Raman behaviors of the devices for 50 cycles in ambient conditions. The behavior of these devices was stable up to 30 cycles, then they started to degrade. The degradation was probably due to the hydration of the ionic liquid in ambient atmosphere, which can be avoided by the passivation of the device. Finally, we found that the infrared behavior of the graphene device was similar for positive and negative bias, which indicated the co-intercalation of anions and cations of the ionic liquid into graphene layers.

## 4. Conclusions

In summary, we demonstrated a flexible multilayer graphene-based device with tunable infrared emissivity. Upon ionic liquid intercalation, the Fermi level shifts to a higher energy level, which blocks optical transition below the Fermi level. Thus, the reflectivity/absorption of graphene increased/reduced, which further reduces the infrared emissivity. Actually, the infrared emissivity of the surface multilayer graphene could be tuned between 0.57 and 0.41 through ionic liquid intercalation/deintercalation. The in-situ Raman and resistivity measurements present a reversible intercalation of anions/cations of the ionic liquid into graphene layers and heavy doping of the graphene layers. These results demonstrate a new strategy for a wide range of applications, such as thermal camouflage, infrared sources, and thermal management.
